# Is ABA the earliest upstream inhibitor of apical dominance?

**DOI:** 10.1093/jxb/erx028

**Published:** 2017-03-23

**Authors:** Thien Q. Nguyen, R.J. Neil Emery

**Affiliations:** 1 Department of Biology, Trent University, Peterborough, Ontario, Canada

**Keywords:** ABA (abscisic acid), Arabidopsis, auxin, axillary bud, branch, competition, phytochrome, R:FR.

## Abstract

This article comments on:

Holalu SV Finlayson SA. 2017. The red light:far red light alters Arabidopsis axillary bud growth and abscisic acid signaling before stem auxin changes. Journal of Experimental Botany 67, 943–952.

**Axillary bud growth – and specifically its release from apical dominance – has been a longstanding and complex area of research. Many phytohormone systems, as well as sugar signaling, have been implicated. Now, Holalu and Finlayson (see pages 943–952) have found compelling evidence that a much less-studied hormone in this context – abscisic acid (ABA) – may, in fact, be one of the first upstream factors regulating apical dominance responses to the R:FR ratio.**

An extraordinary degree of plasticity – the capability to adapt the developmental program to respond to changes in the environment – is one of the most important features of plant development. An outstanding illustration of such developmental plasticity is the control of shoot branching (Domagalska and Leyser, 2011). This key element of plant architecture results from a complex spatio-temporal regulation of axillary bud outgrowth (Rameau *et al.*, 2015). This growth and its release from apical dominance is a process controlled by the elaborate interactions between phytohormones, nutrients and environmental prompts (Barbier *et al.*, 2015; Rameau *et al.*, 2015; Yuan *et al.*, 2015).

For almost a century, the phytohormone auxin has been fundamental to models of apical dominance, particularly how the growing shoot tip suppresses the growth of axillary buds lower down (Mason *et al.*, 2014). Over the years the regulation of bud outgrowth has been described according to two comprehensive models, involving (a) a modification of classic auxin canalization concepts, and (b) the control of second messengers by auxin signaling (Box 1) (Domagalska and Leyser, 2011; Barbier *et al.*, 2015). The former proposes that the inhibition of axillary bud outgrowth is the result of an inability to acquire a polar transport stream, which exports auxin from the bud. This is caused by competition for finite auxin transport capacity in the stem, with the flow through the main shoot being dominant over that from the buds (Domagalska and Leyser, 2011). On the other hand, the latter, alternative model relies largely on the regulatory interactions among auxin and secondary hormonal messengers (Barbier *et al.*, 2015). However, consideration of how auxin interacts with other hormones is complex. Research to date indicates that auxin suppresses cytokinin biosynthesis (Dun *et al.*, 2012), but induces the expression of strigolactone biosynthesis genes in the bud (Mason *et al.*, 2014).

**Box 1. Systemic and axillary bud outgrowth regulatory network models**

In the models shown, sugar and ABA are responsible for initial release of a bud, while auxin, strigolactone (SL) and cytokinin (CK) determine sustained outgrowth. The first model (a) relies on classic auxin canalization concepts. Bud dormancy could be released as a result of (1) the increase in sugar signaling or (2) the decrease in ABA signaling. The establishment of auxin export from an axillary bud to the main stem is a pivotal factor allowing outgrowth. Following decapitation, sugar distribution is disrupted, with the main flow changing to the axillary buds. Consequently, bud dormancy is released. The auxin export stream from the axillary buds restrains the effect of transcriptional activation of BRANCHED 1 (BRC1) and the cell cycle machinery resumes to stimulate bud outgrowth.

The second model (b) is based on the theory that cytokinin and strigolactone are the second messengers. Auxin controls the axillary bud outgrowth by regulating the synthesis of cytokinins and strigolactones, which can go into the axillary bud. Cytokinin promotes the cell cycle machinery and also inhibits BRC1 in axillary buds, while strigolactones stimulate BRC1 expression and subsequently suppress bud outgrowth.

Both theories commonly indicate that the level of R:FR regulates axillary bud outgrowth by regulating PHYB function. A low R:FR ratio inactivates PHYB and thus the PHYB fails to constrain an ABA signal. Meanwhile auxin and strigolactones are dominant signals that promote the BRC1 function in suppressing axillary bud outgrowth. In contrast, high R:FR enhances the PHYB function in suppressing an up-stream ABA signal, releasing axillary bud dormancy. The PHYB activation also suppresses the strigolactone and auxin pathways, leading to inactivation of BRC1. The effect of the level of R:FR on sugar signaling or the interaction between sugar and ABA in the axillary bud release mechanism remains unknown. Adapted from Yuan *et al.* (2015).

**Figure:**
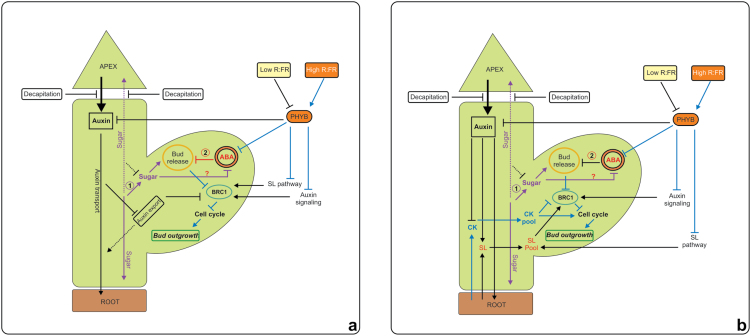

Meanwhile, gibberellins, which are well known for their roles in triggering processes such as flowering and seed germination, need more direct investigation to determine their role in axillary bud outgrowth (Rameau *et al.*, 2015). Some reports on their interaction with strigolactone suggest that increased gibberellin levels could repress axillary bud outgrowth in both strigolactone-dependent and -independent ways (Ferguson and Beveridge, 2009; de Saint Germain *et al.*, 2013). However, an increase in brassinosteroid signaling enhances axillary bud outgrowth by inhibiting strigolactone signaling (Tong *et al.*, 2009; Wang *et al.*, 2013). In yet another study on non-hormonal mechanisms controlling apical dominance, it has been postulated that sugars, rather than auxin, are necessary and sufficient to regulate the very earliest periods of bud outgrowth following decapitation (Barbier *et al.*, 2015; Rameau *et al.*, 2015). The demand for sugars by the intact shoot tip is proposed to override the effects of auxin depletion by blocking the release of axillary bud dormancy (Barbier *et al.*, 2015).

## ABA as an upstream controller of axillary bud outgrowth

Given the extensive history of study, it might at first seem surprising that our understanding of apical dominance remains less than perfectly clear. However, the number of hormonal regulators identified so far does point to a high degree of complexity and, as shown by Holalu and Finlayson (2017), yet more pieces of the puzzle are still being discovered. The experiments presented by these authors use a well-defined and simple system – Arabidopsis cultivated in a split growth chamber and manipulated red:far red (R:FR) wavelengths – to unpack events that occur within just a few hours of bud outgrowth and its related signal switching. They demonstrate that abscisic acid (ABA) levels and associated signaling may play a much more prominent role in the initiation of axillary bud outgrowth than had been thought to be the case. Although the antagonistic effect of ABA on the process has been previously reported (Tucker, 1977; Mader *et al.*, 2003; Reddy *et al.*, 2013), it has not been demonstrated to be such a clear signal of suppression, a full 3 h prior to initiation (Box 2) – it appears to be acting as one of the first upstream controllers of bud suppression caused by low R:FR.

**Box 2. Axillary bud outgrowth**

‘Time lapse’ representation of axillary bud outgrowth, which is concomitant with a reduction in ABA levels.

**Figure:**
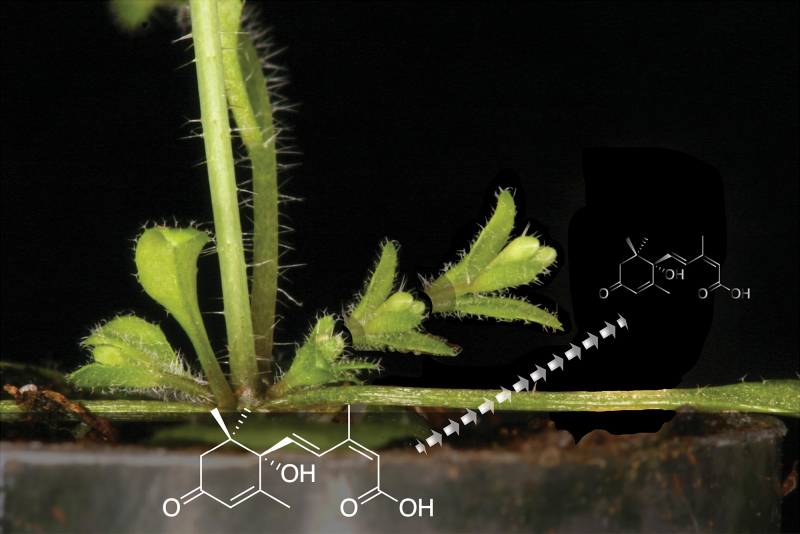

Earlier work related the abundance of bud ABA with the inhibition of branching (Mader *et al.*, 2003), and included research in the context of responses to the R:FR ratio (Tucker, 1977). ABA signaling in these buds was repressed by elevated R:FR, resulting in ABA levels dropping within 12 h of exposure (Reddy *et al.*, 2013). A role for ABA as a regulator of axillary bud outgrowth was characterized using mutants deficient in ABA biosynthesis that displayed inadequate suppression of bud growth in low R:FR (Reddy *et al.*, 2013). More recently, Yao and Finlayson (2015) showed that ABA is able to restrain bud growth under both high and low R:FR and may have a role in suppressing the expression of genes associated with resumption of the cell cycle in dormant buds (Yao and Finlayson, 2015). ABA likewise inhibited the expression of genes associated with the bud autonomous auxin pathway and hindered the accumulation of IAA in the bud; this may be related to the establishment of the bud auxin-exporting stream, which is essential for bud growth (Yao and Finlayson, 2015).

The new paper by Holalu and Finlayson (2017) emphasizes the roles of ABA in axillary bud outgrowth. The authors reveal that as rapidly as 3 h after high R:FR treatment, there is a sustained decline of bud ABA accumulation and signaling, prior to an increase in growth. It is very strong evidence that, unlike auxin, strigolactone or cytokinin, ABA – much less studied in research on apical dominance – may turn out to be one of the earliest upstream factors. Remarkably, they uncover evidence that ABA has a significant negative effect that occurs more rapidly than auxin events.

Could ABA be the earliest upstream regulator in releasing axillary bud outgrowth? Evidence of other early-acting agents must first be considered. In terms of cytokinins (Roman *et al.*, 2016), they have been demonstrated as the initial targets of light in the control of bud outgrowth; the genes of the cytokinin biosynthesis pathway were rapidly up-regulated after 3–6 h of white light exposure and cytokinins accumulated in the nodes within 6 h (Roman *et al.*, 2016). Meanwhile, auxin transport and depletion are much too slow to account for the rapid outgrowth response in garden peas after decapitation (Mason *et al.*, 2014); on the other hand, sucrose alteration was considered to be the earliest response in this scenario, occurring within a 2.5 h timeframe in which statistically significant bud growth was observed (Mason *et al.*, 2014). However, the results of Holalu and Finlayson indicate that sucrose signaling did not appear to be altered as an early response to an increased R:FR ratio. Although it is hard to conclude decisively whether ABA or sucrose might be the first signal to trigger axillary bud outgrowth, the reduction of ABA content is the first response to trigger the bud release given the context of the transition from low to high R:FR. Furthermore, Holalu and Finlayson show that the rapid responses of treated plants to increased R:FR are mediated by changes in their bud ABA physiology, while systemic auxin signaling is necessary for sustained bud repression under low R:FR. These data suggest that the apical dominance effect remains imposed in plants without factors that enhance axillary bud growth and that this is achieved by maintaining a balance of different hormone signals as well as sugar sink strength. In the presence of a bud outgrowth-enhancing factor, such as increased R:FR ratio or apical meristem decapitation, this delicate balance is disrupted by a reduction of ABA or an increase of sugar sink strength which, in turn, results in the release of the axillary bud dormancy. Thus the accumulation of cytokinin and the interactions among auxin, strigolactone and cytokinin are more involved in the transition from axillary bud release to sustained bud growth and elongation.

## Perspectives

The regulation of apical dominance and axillary bud outgrowth is the result of a complex physiological network of signals, in which significant roles are currently believed to be reserved for auxin, cytokinin, strigolactone and sugar, as well as environmental signals such as light quality and soil nutrient status (Domagalska and Leyser, 2011; Mason *et al.*, 2014; Roman *et al.*, 2016). In a web of influencers, a significant change of any one factor will generate change in others (e.g. see Murphy, 2015, and references therein).

Holalu and Finlayson’s work should inspire other researchers to start investigating in the same area. Over the years, ABA has not gained much attention in studies on apical dominance, though much research has hinted at its importance – it should now be firmly added as an agent within the regulatory network. New follow-up studies taking a more manipulative, functional genomic approach would be especially useful. Simulation modeling could also be productive. To obtain a full picture of the collective effects of the regulatory network in maintaining apical dominance, and in releasing and sustaining axillary bud outgrowth, much more work will be needed to fill the gaps in our understanding of this longstanding puzzle.
